# Narrative review investigating the nephroprotective mechanisms of sodium glucose cotransporter type 2 inhibitors in diabetic and nondiabetic patients with chronic kidney disease

**DOI:** 10.3389/fendo.2023.1281107

**Published:** 2023-12-20

**Authors:** Emma S. Speedtsberg, Martin Tepel

**Affiliations:** ^1^ Institute of Molecular Medicine, Cardiovascular and Renal Research, University of Southern Denmark, Odense, Denmark; ^2^ Institute of Clinical Medicine, University of Southern Denmark, Odense, Denmark; ^3^ Department of Nephrology, Odense University Hospital, Odense, Denmark

**Keywords:** diabetic chronic kidney disease, nondiabetic chronic kidney disease, sodium glucose cotransporter type 2 inhibitors, nephroprotection, pathophysiologic mechanisms

## Abstract

**Background and aims:**

Outcome trials using sodium glucose cotransporter type 2 inhibitors have consistently shown their potential to preserve kidney function in diabetic and nondiabetic patients. Several mechanisms have been introduced which may explain the nephroprotective effect of sodium glucose cotransporter type 2 inhibitors beyond lowering blood glucose. This current narrative review has the objective to describe main underlying mechanisms causing a nephroprotective effect and to show similarities as well as differences between proposed mechanisms which can be observed in patients with diabetic and nondiabetic chronic kidney disease.

**Methods:**

We performed a narrative review of the literature on Pubmed and Embase. The research string comprised various combinations of items including “chronic kidney disease”, “sodium glucose cotransporter 2 inhibitor” and “mechanisms”. We searched for original research and review articles published until march, 2022. The databases were searched independently and the agreements by two authors were jointly obtained.

**Results:**

Sodium glucose cotransporter type 2 inhibitors show systemic, hemodynamic, and metabolic effects. Systemic effects include reduction of blood pressure without compensatory activation of the sympathetic nervous system. Hemodynamic effects include restoration of tubuloglomerular feedback which may improve pathologic hyperfiltration observed in most cases with chronic kidney disease. Current literature indicates that SGLT2i may not improve cortical oxygenation and may reduce medullar oxygenation.

**Conclusion:**

Sodium glucose cotransporter type 2 inhibitors cause nephroprotective effects by several mechanisms. However, several mediators which are involved in the underlying pathophysiology may be different between diabetic and nondiabetic patients.

## 1 Introduction

Chronic kidney disease (CKD) affects around one in ten people worldwide and is largely a contributor to mortality and reduced quality of life (1–4). Because the number of people living with risk factors for kidney disease increases, the number of patients suffering from CKD and the number of patients dying from the disease, continues to rise (1–4). The most common causes of CKD include diabetes mellitus (DM), hypertension, and glomerulonephritis (GN) (4). The definition of CKD includes structural or functional changes with persistence of at least 3 months. The diagnosis is based on either the presence of kidney damage markers, for example, albuminuria, or a decreased glomerular filtration rate (GFR) (4). Until recently, blockade of the renin angiotensin aldosterone system (RAAS) with angiotensin converting enzyme inhibitors (ACEi) and angiotensin receptor blockers (ARB) have been cornerstones to slow progressive decline of kidney function (5). Several large outcome trials have consistently shown the nephroprotective potential of sodium glucose cotransporter type 2 inhibitors (SGLT2i) in diabetic patients with CKD (6–9).

The recent KDIGO clinical practice guideline for diabetes management in chronic kidney disease recommends treatment of patients with type 2 diabetes mellitus, chronic kidney disease, and an estimated glomerular filtration rate more the 20mL per 1.73m2 with SGLT2i (10). The authors noticed that start of SGLT2i treatment may cause a reversible decrease in the eGFR and was not an indication for discontinuation (10). Due to lacking evidence administration of SGLT2i does not apply to kidney transplant recipients (10). The EMPA-Kidney trial showed that administration of SGLT2i, empagliflozin, to patients with kidney disease for 2 years significantly reduced the composite endpoint, progression of kidney disease or death from cardiovascular causes. The outcome was observed in 13.1% (432 of 3304 patients) in the empagliflozin group and in 16.9% (558 of 3305 patients) in the placebo group (hazard ratio, 0.72, 95% CI 0.64 to 0.82) (11). It is important to mention that the results were consistent among patients with or without diabetes (11). The authors also indicated that SGLT2i, empagliflozin, was beneficial in patients with an eGFR less than 30 ml per minute or a low urinary albumin-to-creatinine ratio (11).

A recent publication presented the American Diabetes Association (ADA) and Kidney Disease Improving Global Outcomes (KDIGO) consensus statement that SGLT2i is recommended for patients with diabetes mellitus type 2, chronic kidney disease, and an eGFR more than 20 ml/min/1.73m2 (12). This recommendation was based on strong evidence from large outcome trials that SGLT2i may reduce the progression of chronic kidney disease, heart failure and atherosclerotic cardiovascular disease in these patients (12). The authors indicate that SGLT2i reduce intra-glomerular pressure (12).

**Figure 1:**
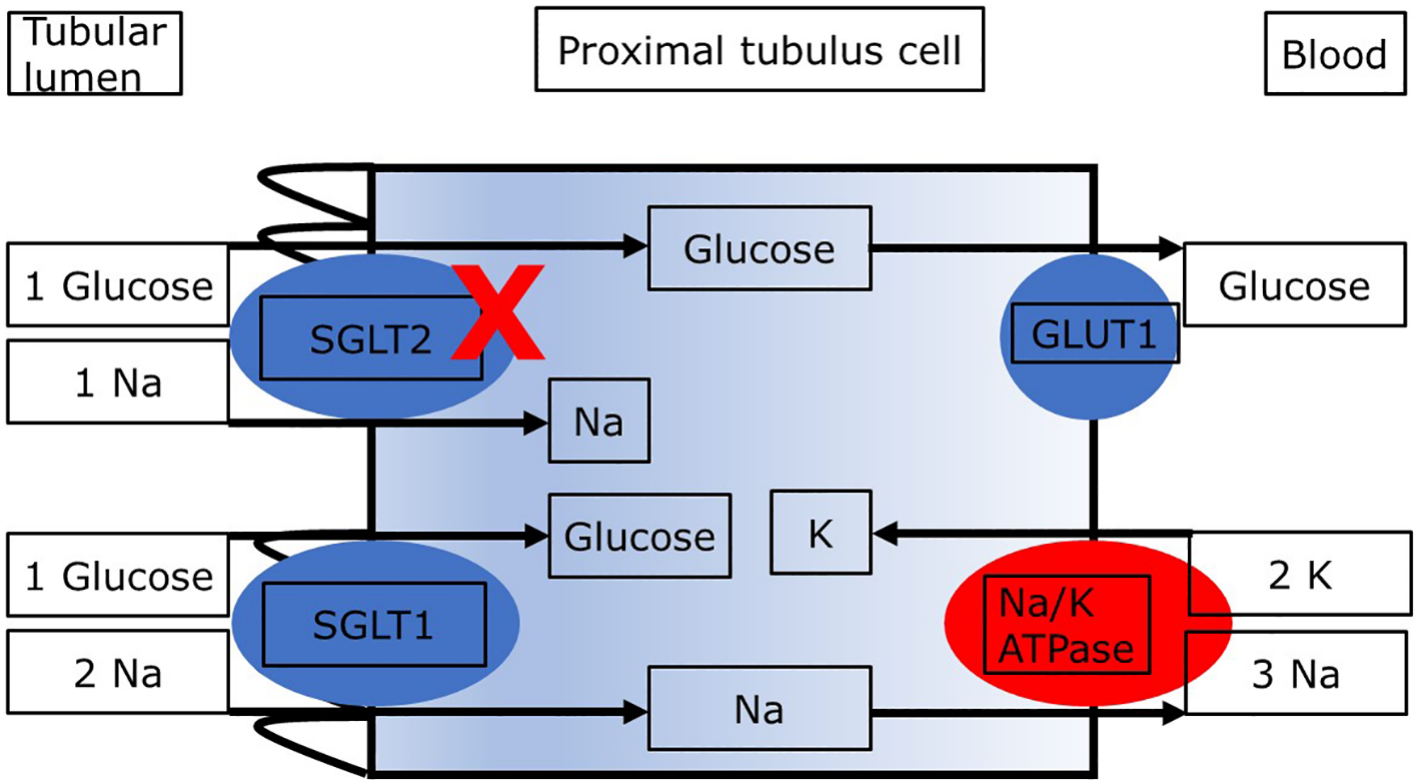
Proximal tubule cell with transporters relevant to the reabsorption of glucose. Blockade of SGLT2 results in increased delivery of glucose and sodium to downstream segments and loss in the urine. ATP, adenosine triphosphate; GLUT2, glucose transporter 2; SGLT1, sodium glucose cotransporter type 2. The sodium:glucose coupling ratio is 1:1 for SGLT2 and it is 2:1 for SGLT1. The different sodium:glucose coupling ratios may impact renal energy expenditure with SGLT2i.

As shown in **Figure 1**, the reabsorption of filtered glucose from the tubular lumen is caused by two transporters located apically on the proximal tubule cells (13). SGLT2 is situated in the S1 segment, whereas sodium glucose cotransporter type 1 (SGLT1) is situated in the distal S3 segments of the proximal tubule located at the corticomedullary junction (13). These transporters work in conjunction with the basolateral sodium potassium pumps, which uses energy in the form of adenosine triphosphate (ATP) to create an electrochemical gradient. The transport of glucose to the bloodstream is facilitated passively by glucose transporter type 2 (GLUT2). Since SGLT2 transporters are responsible for the uptake of filtered glucose in cotransport with sodium in a 1:1 ratio, inhibition of SGLT2 causes glucosuria and increases natriuresis (14). Which mechanisms may link the increased loss of glucose and sodium with kidney protection and lower mortality? Several mechanisms regarding the nephroprotective effect in diabetic as well as non-diabetic patients with CKD have been proposed. The objective of this review is to describe the main mechanisms underlying the nephroprotective effect of SGLT2i in patients with diabetic nephropathy. The second aim is to demonstrate similarities and differences in patients with diabetic and nondiabetic CKD regarding these mechanisms. We present the following article in accordance with the Narrative Review reporting checklist.

## 2 Materials and methods

### 2.1 Search strategy, study selection and eligibility criteria

**Figure 2:**
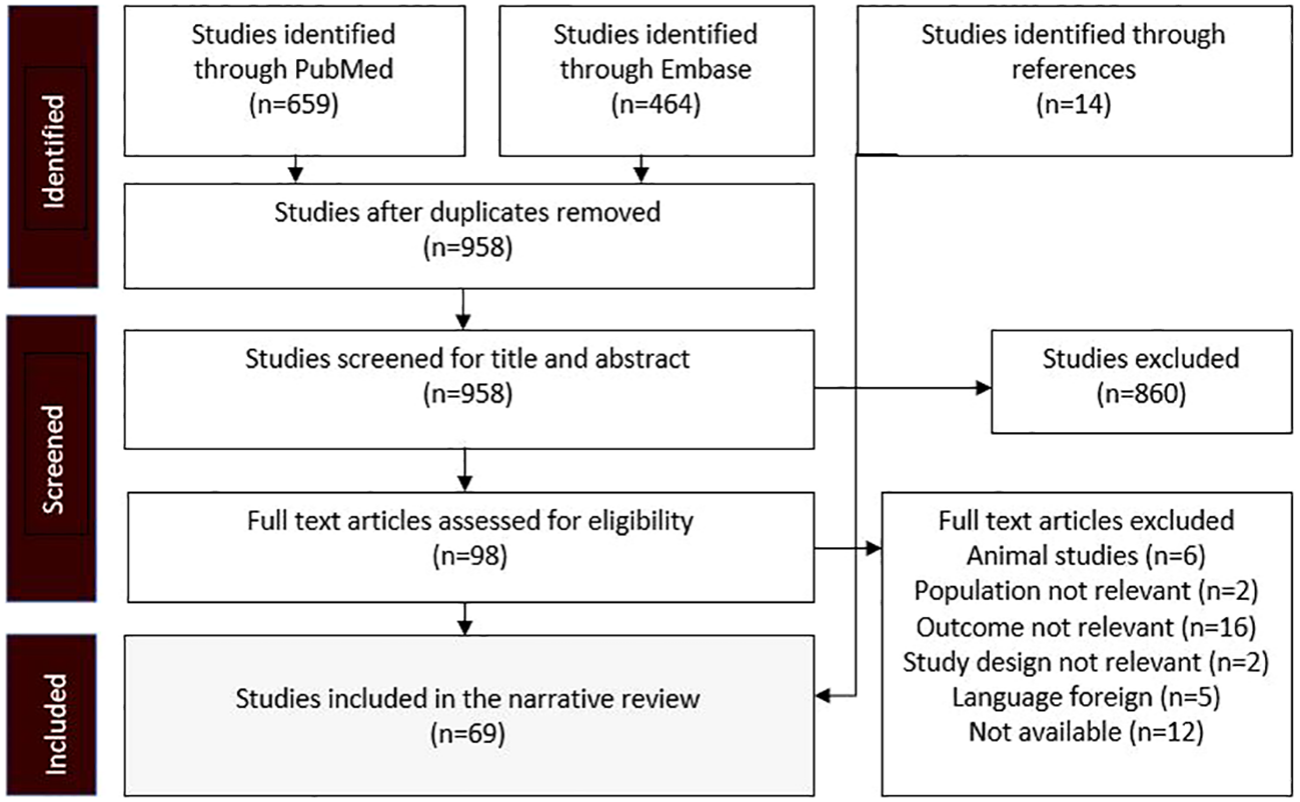
Flow diagram showing the process whereby studies were identified, screened, and included. The exclusion criteria were predetermined before the research.

The research strategy summary is given in **Table 1**. The databases Pubmed and Embase were searched for original research and review articles from start until March, 2022. Based on the objectives, relevant blocks were constructed and terms and synonymous for each block were identified (**Table 2**). The main words were “chronic kidney disease, sodium glucose transporter 2 inhibitor, and mechanism”. Synonyms were found from articles by the initial unstructured research, and by selecting “show index” for each word in the search function in Pubmed. Quotes were used to ensure that the words were not searched for individually and truncation was used to allow the word to have multiple endings. The individual words within each block were combined with OR, while each block was combined with AND. From this, a search string for each database was constructed, including both free text terms and keywords. The studies identified from the two databases, were imported into the reference system Endnote (Clarivate Analytics, Philadelphia, USA) and duplicates were removed automatically. Then they were exported to the screening tool Rayyan (Rayyan Systems Inc, Cambridge, England) and additional duplicates were removed manually. The studies were screened for title and abstract, and exclusion was done by the authors if words from the three blocks were not included, or if the study was made in animals only. The remaining full text articles were screened and included if they did not meet the following exclusion criteria: animal studies only, the population did not have kidney disease, the article was a comment, the language was not Danish or English (**Figure 2**). Additional studies were identified through included references.

**Table 1:** Research strategy summary.

Items	Specification
Date of Search (specified to date, month and year)	March 4-15, 2022.
Databases and other sources searched	Pubmed, Embase and reference lists.
Search terms used (including MeSH and free text search terms and filters) Note: please use an independent supplement table to present detailed search strategy of one database as an example.	Free text terms: chronic kidney disease, CKD, chronic kidney disease, “chronic impaired kidney function”, “chronic renal dysfunction”, chronic renal impairment, chronic renal disease, chronic renal failure, chronic renal insufficiency, sodium glucose transporter 2 inhibitor, SGLT2i, mechanism, physiology, hemodynamic, metabolic, molecular. Keywords: chronic kidney failure, sodium glucose cotransporter 2 inhibitor, drug mechanism, physiology.
Timeframe	Publications from 2005-2022.
Inclusion and exclusion criteria (study type, language restrictions etc.)	Inclusion: title and abstract including words from all three blocks. Exclusion: animal studies, population not relevant, outcome not relevant, study design not relevant, language foreign, not available.
Selection process (who conducted the selection, whether it was conducted independently, how consensus was obtained, etc.)	The databases were searched independently and the agreements by two authors were jointly obtained.
Any additional considerations, if applicable	Population could be patients with various causes of chronic kidney disease, including patients with type 1 and type 2 diabetes mellitus and patients without diabetes mellitus.

**Table 2:** The table shows the tree blocks which were constructed based on the objectives, with terms and synonymous relevant to each block.

Block: population	Block: intervention	Block: outcome
**Chronic kidney disease**	Sodium glucose transporter 2 inhibitor	Mechanism
**CKD**	SGLT2i	Physiology
**Chronic kidney failure**		Hemodynamic
**Chronic impaired kidney function**		Metabolic
**Chronic renal dysfunction**		Molecular
**Chronic renal impairment**		
**Chronic renal disease**		
**Chronic renal failure**		
**Chronic renal insufficiency**		

## 3 Results

The nephroprotective effects of SGLT2i are summarized in **Table 3**, and have been attributed to systemic, hemodynamic, and metabolic mechanisms. The details extracted from references included in this review are given in **Table 4**. Some of the mechanisms may affect risk factors which are observed more often in diabetic patients, whereas others may target features common to all patients with CKD.

**Table 3:** Parameters/markers/systems affected, changes from baseline, and the probable mechanisms of SGLT2 inhibitors.

Parameter/marker/system	Change from baseline	Probable mechanisms according to literature
Plasma glucose levels	Reduced	Glucosuria
Body weight	Reduced	Caloric loss results in reduced body weight.
Systolic and diastolic blood pressure	Reduced	Natriuresis results in reduced extracellular volume and thus lowers systolic and diastolic blood pressure
Plasma uric acid	Reduced	Glucose and uric acid compete with the same GLUT9 transporter. When higher glucose levels are available in the tubular lumen, the transport of uric acid into the blood is reduced.
Sympathetic nerve system	Reduced	No compensatory increase in heart rate despite reduction of extracellular volume. Mediators are only partly known.
Tubuloglomerular feedback	Increased	Increased delivery of sodium to macula densa cells, which is amplified by downregulation of the sodium-proton-exchanger 3 (NHE3), stimulates the release of vasoactive mediators. These affect the glomerular arterioles, thus ameliorating glomerular hyperfiltration.
Renin-angiotensin-aldosterone-system (RAAS)	Increased	The increased release if renin is due to reduced effective vascular volume. In the presence of RAAS blockade with ACEi of ARB several mediators are directed from the vasoconstrictive and pro-inflammatory classical pathway to the vasodilating and anti-inflammatory alternative pathways.
Ketogenesis	Increased	Stimulation of starvation like stages induce transcription factors and decrease insulin secretion. This stimulates ketogenesis. Ketones are energy efficient fuels producing more ATP from oxygen compared to free fatty acids.
Hematocrit	Increased	Partly due to reduced effective vascular volume and enhanced erythropoiesis. That causes compensatory upregulation of SGLT1 in the medulla which results in local hypoxia and stimulates the transformation of erythropoietin-producing fibroblasts.
Renal tissue oxygenation	Cortex: No change. Medulla: Reduced	Reduced oxygen demand in the cortex may be due to reduced sodium transport and increased utilization of ketones. Increased oxygen delivery is partly explained by elevated hematocrit. Furthermore, intensified medullary hypoxia due to reabsorption of glucose and sodium and tubular segments downstream the location of SGLT2.
Autophagia	Increased	Ketones upregulate transcription factors which promote autophagia.
Cellular inflammatory response and inflammasome	Decreased	Ketones inhibit histone deacylases and inflammasomes.
Renal fibrosis	Decreased	On one hand, improved renal cortical oxygenation results in decreased inflammation and fibrosis through downregulation of hypoxia inducible transcription factors and others. On the other hand, there is enhanced medullary hypoxia. Enhanced medullary transport leading to medullary hypoxia occurs in medullary thick limbs in the inner- and outer stripe of the outer medulla, with an increased activity of sodium-potassium-ATPase in the basolateral membrane and sodium-potassium-2-chloride cotransporter at the apical membrane.

**Table 4:** Studies included in this narrative review.

A: Original research articles		
Author (Ref)	Publication year Study design	Main findings
***Cherney et al.*** (15)	2020 RCT Dpagliflozin	No reduction in proteinuria. Reversible decline of eGFR.
***Heerspink et al.*** (16)	2016 RCT Dapagliflozin	Reduction in albuminuria independent of changes in HbA1c, systolic blood pressure, bodyweight and eGFR.
***Nojima et al.*** (17)	2020 RCT Tofogliflozin	Lowered heart rate, improved insulin resistance.
***Cherney et al.*** (18)	2014 Dapagliflozin	Attenuation of renal hyperfiltration.
***Van Bommel et al.*** (19)	2020 RCT Dapagliflozin	Reduction of GFR in patients with DM.
***Li et al.*** (20)	2020 Canagliflozin	Reduced urinary pH indicate blockade of sodium-proton-exchanger 3
***Rajasekeran et al.*** (21)	2018 Dapagliflozin	Decreased expression of SGLT2 mRNA in patients with FSGS
***Antlanger et al.*** (22)	2022 RCT Empagliflozin	Empagliflozin on top of an Angiotensin-Converting-Enzyme-Inhibitor (ACEi) induced activation of the vasodilating and anti-inflammatory alternative pathways in diabetic patients.
***Yoshimoto et al.*** (23)	2017 Case study	Limited effect of SGLT2i to activate RAAS in diabetic patients.
***Heise et al.*** (24)	2016 RCT Empagliflozin	No changes in plasma renin or serum aldosterone.
***Heerspink et al.*** (25)	2013 RCT Dapagliflozin	Increase of hematocrit and hemoglobin.
***Laursen et al.*** (26)	2021 RCT Dapagliflozin	Reduciton of renal resistance.
***Liu et al.*** (27)	2021 RCT Ertugliflozin	Reduction of kidney injury molecule 1.
***Dekkers et al.*** (28)	2018 RCT Dapagliflozin	Reducito of kidney injury molecule 1.
***Wang et al.*** (29)	2017 Case control	Increased expression of SGLT2 mRNA and protein in biopsies from patients with type 2 DM and CKD.
***Rahmoune et al.*** (30)	2005 Case control	Increased expression of SGLT2 mRNA and protein, in renal tubular cells in urine samples from diabetic patients.
***Solini et al.*** (31)	2017 Case control	Increased expression of SGLT2 mRNA and protein in nondiabetic patients.
***Sridhar et al.*** (32)	2019 Cross control	Reduced renal SGLT2 mRNA expression in diabetic patients.

B: Reviews including diabetic patients		
Author (Ref)	Publication year	Main findings
***Vallon et al.* (** 14 **)**	2021	Upregulation of SGLT2 in diabetic CKD. Upregulation of Sodium-Hydrogen-Exchanger type 3 (NHE3).
***Zhao et al.*** (33)	2018	Reduction of plasma uric acid.
***Gillard et al.*** (34)	2020	Hyperglycemia increases urinary inflammatory markers and may lead to RAAS activation.
***Rocha et al.*** (35)	2018	Reduction of blood pressure reduction without compensatory increase in heart rate.
***Liu et al.*** (36)	2022	Restoration of TGF.
***Kanduri et al.*** (37)	2020	Restoration of TGF. Downregulation of NHE3.
***Packer* (** 38 **)**	2021	Upregulating of oxygen delivery and downregulation of oxygen demand.
***Gnudi et al.*** (39)	2016	Restoration of TGF. Increased vasodilating and anti-inflammatory alternative pathways.
***Packer* (** 40 **)**	2020	Stimulation of transcription factors resulting in ketogenesis, erythropoiesis and autophagia.
***Brown et al.*** (41)	2019	Reduced blood glucose, reduced secretion of insulin and increased secretion of glucagon.
***Ito et al.*** (42)	2018	Reduced oxygen consumption due to ketogenesis.
***Hesp et al.*** (43)	2020	Enhanced energy consumption through upregulation of SGLT2.
***Packer* (** 44 **)**	2020	Reduction of blood and urine biomarkers of autophagic proteins.
***Yaribeygi et al.*** (45)	2018	Reduction of the inflammatory response.
***Packer* (** 46 **)**	2020	Downregulation of SGLT2 and NHE3. Activation of transcription factors promoting autophagia.
***Hattori* (** 47 **)**	2021	Restoration of TGF. Increased keton bodies. Inhibition of histone deacetylases and inflammasomes.
***Gilbert* (** 48 **)**	2014	Reduction of plasma uric acid.
***Cherney et al.*** (49)	2014	Better blood pressure control.
***Heerspink et al.*** (50)	2016	Elevated RAAS metabolites in urine and blood from the vasoconstrictive and pro-inflammatory classical and vasodilating and anti-inflammatory alternative pathways.
***Satirapoj*** (51)	2017	Reduction of inflammatory, oxidative, and fibrotic markers.
***Van Bommel et al.*** (52)	2017	Multiple mechanisms underlying the nephroprotective effects.
***Thomas et al.*** (53)	2018	Changes in solute, water and energy balance in the proximal tubule following SGLT2 inhibition.
***Tsimihodimos et al.*** (54)	2018	Improvements in several pathways and metabolic variables.
***Alicic et al.*** (55)	2019	Reduced glomerular hyperfiltration and hypertension.
***Kuriyama*** (56)	2019	Attenuation of renal fluid congestion.
***Sarafidis et al.*** (57)	2019	Amelioration of glomerular hyperfiltration.
***Thomson et al.*** (58)	2019	Reduction of SGLT2 veractivity.
***Hou et al.*** (59)	2020	Restoration of TGF. Arteriole constriction by adenosine and efferent arteriole dilatation by prostaglandins.
***Kashihara et al.*** (60)	2020	Multiple mechanisms.
***Packer* (** 61 **)**	2020	Upregulation of “starvation” transcription factors with increased ketogenesis.
***Schnell et al.*** (62)	2020	Restoration of TGF.
***Zelniker et al.*** (63)	2020	Multiple hemodynamic and metabolic changes.
***Lee et al.*** (64)	2021	Multiple mechanisms including hemodynamic and non-hemodynamic mechanisms.
***Onyali et al.*** (65)	2021	Benefits are independent of glycemic control.
***Puglisi et al.*** (66)	2021	Increased angiotensin1-7.
***Leoncini et al.*** (67)	2021	Multiple mechanisms.
***Din et al.*** (68)	2021	Multiple mechanisms.
***Provenzano et al.*** (69)	2021	Restoration of TGF.
***Srinivas et al.*** (70)	2021	Reduction of interstitial fluid instead
***Castañeda et al.*** (71)	2021	Attenuation of hyperfiltration.
***Takata et al.*** (72)	2021	Pleiotropic effects.

C: Reviews including diabetic and nondiabetic patients		
Author (Ref)	Publication year	Main findings
***Pollock et al.*** (73)	2021	Multiple mechanisms.
***Bailey* (** 74 **)**	2019	Reduction of plasma uric acid. Glucose and uric acid compete with the same transporter.
***Yip et al.*** (75)	2022	Reduction of serum uric acid.
***Dekkers et al.*** (76)	2018	Amelioration of glomerular hyperfiltration.
***Nayak et al.*** (77)	2021	Inhibition of NHE3 plays an essential role in TGF activation in nondiabetics. Increased plasma and urinary ketones in nondiabetic patients.
***Dekkers et al.*** (78)	2020	Restoration of TGF in type 1 DM.
***Rajasekeran et al.*** (79)	2017	Natriuretic effects extend to nondiabetic CKD.
***Ekanayake et al.*** (80)	2022	Lipolysis and ketogenesis. Ketones improve renal tissue oxygenation and show anti-inflammatory and antifibrotic properties.
***Herrington et al.*** (81)	2021	Reduction of intraglomerular hypertension in CKD.
***Oguz et al.*** (82)	2021	Amelioration of single nephron GFR.

### 3.1 Systemic mechanisms

Due to glucosuria and natriuresis, a modest reduction in hemoglobin A1c (HbA1c), bodyweight, as well as systolic and diastolic blood pressure have been observed in several studies in diabetic patients with CKD. In nondiabetic patients with CKD, Cherney et al. showed a reduction in bodyweight, but no significant changes in HbA1c or blood pressure (15). Since overweight as well as increased blood pressure are known risk factors for progression of CKD, these mechanisms likely confer nephroprotection by SGLT2i. Heerspink et al. found, that a reduction in albuminuria in patients with type 2 DM treated with a SGLT2i appeared to be independent of changes in HbA1c, bodyweight and systolic blood pressure (16). It should be noted that the effect on glucosuria is attenuated in patients with reduced kidney function, while blood pressure lowering is quite consistent across different levels of kidney function (73). Large outcome studies showed that sodium glucose cotransporter type 2 inhibitors (SGLT2i) reduce systolic/diastolic blood pressure by approximately 4 mmHg/2 mmHg (83). Compared to placebo mean reduction of 24-hour diastolic blood pressure were 1.0 mmHg, 1.3 mmHg, and 4.8 mmHg in patients with eGFR more than 90 ml/min per 1.73 m2, eGFR between 90 and 60 ml/min per 1.73 m2, and eGFR between 60 and 30 ml/min per 1.73 m2, respectively (84). SGLT2i do not act as osmotic diuretics. But low-level ketoacidosis which is observed after administration of SGLT2i can reduce blood pressure (80). In the Dahl salt-sensitive rat model of hypertension, the co-administration of β-hydroxybutyrate reduced elevated blood pressure in a salt-rich diet (85).

Reduced plasma uric acid is a consequence of increased glucose in the tubular lumen, which is taken up by the same glucose transporter type 9, at the expense of uric acid. Results have been conflicting, as to whether lowering of plasma uric acid provides nephroprotection. Some studies indicate that hyperuricemia is associated with increased risk of kidney disease in patients with type 1 DM (86, 87) and in patients with type 2 DM (74). Other studies showed that interventions to reduce plasma uric acid could retard the progression of CKD (88, 89). In contrast, other studies indicated that uric acid may not be directly involved in the development of CKD in diabetic patients, but is a downstream marker of kidney damage (90), hence trials using febuxostat and allopurinol failed to show a large nephroprotective effect (91, 92). Zhao et al. found that the reduction of plasma uric acid is attenuated with lower estimated glomerular filtration rate (eGFR) (33). These studies concluded that uric acid lowering alone may not provide nephroprotection, but they do not exclude that in a combination with other mechanisms offered by SGLT2i, the observed lowering of plasma uric acid may have an additional beneficial effect (34).

Elevated concentrations of plasma uric acid have been associated with increased risk of development and progression of CKD in nondiabetic patients, and interventions to reduce uric acid, may contribute to the nephroprotective effect in these patients (74). A recent meta-analysis of 43 randomized controlled trials indicated that SGLT2i reduced plasma uric acid levels in both diabetic and nondiabetic patients (75). However, the effect may be smaller in nondiabetic patients, because the concentration of plasma uric acid is generally lower, and the uricosuric effect may be smaller due to the lower filtered glucose, capable of competing with the glucose transporter type 9 (74). The beneficial effects of SGLT2i may be due to the fact that fractional uric acid excretion was strongly correlated to fractional glucose excretion (93).

The sympathetic nervous system (SNS) is not activated upon SGLT2 inhibition, which is proved by the ability of SGLT2i to reduce systolic and diastolic blood pressure without a compensatory increase in heart rate (35). That may be due to lower adipose tissue insulin resistance (17). Furthermore, SGLT2i, dapagliflozin, may directly attenuate the sympathetic response (94).

### 3.2 Hemodynamic mechanisms

The restoration of the tubuloglomerular feedback (TGF) mechanism has been considered to be an outstanding explanation why SGLT2i offers nephroprotection, because it targets common steps in the pathogenesis of CKD, in particular the glomerular hyperfiltration (76). Vasodilation of the afferent arteriole which can be observed in patients with diabetes mellitus or in patients with high protein intake causes glomerular hyperfiltration (95). Activation of renin-angiotensin-aldosterone system leads to efferent arteriolar vasoconstriction which causes glomerular hypertension (95). Furthermore, glomerular hyperfiltration is a consequence of reduced number of nephrons in CKD, resulting in a compensatory increase in glomerular filtration in the remaining nephrons (4).

Large outcome trials consistently showed a significant initial decline of eGFR following administration of SGLT2i (5–9). By blocking the reabsorption of glucose and sodium, an increased amount of sodium can be observed at the macula densa cells, which leads to the release of nucleosides finally affecting the tone of the afferent arteriole. Different mediators may contribute and different effects on glomerular vascular tone have been proposed, depending on the cause which resulted in hyperfiltration.

In patients with type 1 DM, Cherney et al. measured eGFR using inulin clearance and renal plasma flow using paraaminohippurate clearance together with circulating levels of RAAS and nitric oxide. These values were measured under clamped euglycemic and hyperglycemic conditions at baseline and at the end of treatment with SGLT2i. Cherney et al. observed that attenuation of hyperfiltration was accompanied by decreased renal plasma flow, increased renal vascular resistance and no changes of vasodilators, including urinary prostaglandins and nitric oxide (18). They suggested that SGLT2i affect TGF and afferent arteriole constriction, and mentioned adenosine as a major vasoconstrictor involved. In patients with type 2 DM, van Bommel et al. showed that attenuation of hyperfiltration was associated with increased levels of urinary adenosine and prostaglandins but no increase in renal vascular resistance (19). That may point to high baseline RAAS inhibition and high afferent arteriole constriction at baseline, which may limit further vasoconstriction. Adenosine has several receptors, and binding to adenosine A1 receptors on the efferent arteriole likely causes vasodilation, which may be reinforced by increased production of vasodilating prostaglandins (36).

In nondiabetic patients, Cherney et al. demonstrated the ability of SGLT2i to attenuate hyperfiltration, as indicated by the initial decline of eGFR during treatment (15). They did not detect a reduction in albuminuria which has been shown in previous studies in diabetic patients (15). One reason could be the short duration of treatment. Another reason could be differences in underlying disease pathologies because some sources of proteinuria are less responsive to changes in eGFR (15). Finally, an explanation could be a weaker activation of macula densa cells in comparison to diabetic patients, because the amount of filtered glucose is already lower in nondiabetic patients, and the initial decline of eGFR leads to further lowering of glucose and sodium delivery to macula densa cells. Hence TGF might not be activated sufficiently to reduce proteinuria in nondiabetic patients who already have a low GFR (15). They did not observe an association between the initial decline of eGFR and changes in adenosine or prostaglandins. Therefore, other vasoactive mediators, i.e., endothelin and nitric oxide could be involved in nondiabetic patients (15).

Downregulation of sodium hydrogen exchanger type 3 and thus reduced reabsorption of sodium may also contribute to restoration of TGF (37). Downregulation of sodium hydrogen exchanger type 3 may occur because the activities of sodium hydrogen exchanger type 3 and SGLT2 are closely linked, i.e., SGLT2 presumably increases the activity of sodium hydrogen exchanger type 3 (96). This was demonstrated clinically by reduced urine pH following SGLT2i, due to the urinary loss of hydrogen (20). By using a mathematical model of renal function and volume homeostasis in combination with clinical data, it has been predicted that inhibition of apical proximal tubule sodium hydrogen exchanger type 3 is required for the natriuretic effect induced by SGLT2i in humans (97). Since sodium and chloride remain the sole solute sensor for macula densa cells, downregulation of sodium hydrogen exchanger type 3 may also play an essential role in TGF activation in nondiabetic patients (77).

Given the TGF mechanism, it is possible that several kidney diseases might benefit from SGLT2i (78). Diseases like obesity induced nephropathy, hypertensive nephropathy as well as several types of GN are characterized by renal hemodynamic changes including glomerular hypertension and hyperfiltration (79). They therefore share a common step in the pathogenesis leading to CKD, namely damage and loss of nephrons and thus hyperfiltration in remaining nephrons, creating a vicious cycle. Kidney diseases characterized by glomerular hyperfiltration will likely benefit from SGLT2 inhibition via restoration of TGF, but the mediators involved likely are different in various diseases.

Despite being an important and well-studied mechanism, restoration of TGF may only partly explain the large nephroprotective effect of SGLT2i. Rajasekeran et al. questions whether this mechanism is central in all subtypes of CKD, as they failed to observe any favorable renal hemodynamic alterations or attenuation in albuminuria in patients with focal segmental glomerulosclerosis (FSGS) after administration of an SGLT2i (21). This could be due to the loss of transporters in these patients. In addition, Heerspink et al. showed that in patients with type 2 DM, a reduction in albuminuria was maintained even after adjustment for changes in eGFR, suggesting that other mechanisms may be involved (16). To support the contribution from other mechanisms, Packer claims that patients with very low filtration rates still benefit from SGLT2i treatment, despite the fact that amelioration of hyperfiltration by SGLT2i is probably limited in these patients (38).

Blood pressure is affected by both natriuresis as well as the Renin-Angiotensin-Aldosterone-System.

An increased sodium delivery to the macula densa activates the tubuloglomerular feedback increasing the resistance in the afferent arteriole (76). In contrast, the Renin-Angiotensin-Aldosterone-System mainly affects the resistance in the efferent arteriole. Furthermore, SGLT2i-induced activation of the tubuloglomerular feedback may reduce glomerular filtration by affecting the afferent arteriole thereby reducing excreted sodium.

Reduced effective circulating volume, which may be a consequence of SGLT2i, results in increased release of renin and thus production of angiotensin I from angiotensinogen. In the presence of RAAS blockade, by an ACEi or an ARB, angiotensin I is converted to angiotensin 1-7 by angiotensin converting enzyme 2. These are mediators of the vasodilating and anti-inflammatory alternative pathways, and the importance of these mediators as key opposing effectors to angiotensin II has been well established (98). Antlanger et al. reported elevated plasma angiotensin I and angiotensin 1-7 after administration of an SGLT2i on top of an ACEi in patients with type 2 DM (22). They propose that ACEi cannot fully reverse CKD progression due to normalization of angiotensin II levels after long-term therapy, a phenomenon termed “ACEi escape” (22). They conclude that suppression of angiotensin II with RAAS blockade in combination with stimulation of the vasodilating and anti-inflammatory alternative pathways by SGLT2i could therefore be of importance (22). The presumed theory is that angiotensin II induces vasoconstriction and inflammation while angiotensin 1-7 promote vasodilatation and have anti-inflammatory properties (39). In contrast, Yoshimoto et al. conclude that the ability of SGLT2i to activate RAAS in patients with type 2 DM is limited (23). They found no increase in urinary angiotensinogen during treatment with different SGLT2i. In addition, Heise et al. did not observe any changes in plasma renin or serum aldosterone during SGLT2i (24).

### 3.3 Metabolic mechanisms

Continuous glucosuria, and thereby loss of calories through the urine, simulates a starvation like state resulting in a metabolic shift from glycolysis to lipolysis and ketogenesis (80). This is presumably due to upregulation of several transcription factors normally induced in the fasting state (40). In addition, the decline in blood glucose leads to reduced secretion of insulin and an increased secretion of glucagon (41). In addition, ketogenesis is associated to direct upregulation of energy deprivation sensors like AMPK (adenosine monophosphate-activated kinase) and SIRT1 (Sirtuin 1) (99).

Ketone bodies are an efficient fuel substrate because they generate more ATP for the same amount of oxygen compared to free fatty acids (FFA) (80). Ketogenesis could therefore probably contribute to improved renal tissue oxygenation, by reducing renal oxygen consumption (42). Packer claims, that it is unlikely that the ability of SGLT2i to increase ketone bodies, is responsible for the nephroprotective effect in diabetic nephropathy, since circulating levels of ketone bodies are already increased in diabetic patients in the absence of treatment (38). During treatment with SGLT2i, a doubling of ketone bodies in plasma has been observed in nondiabetic patients similar to what is found in diabetic patients (100). This is accompanied by increased levels of ketone bodies and metabolites from ketogenesis in the urine (77). Because impaired tissue oxygenation plays an equally crucial role in progression of CKD of various subtypes, its reversal may be important (43). Due to this, the beneficial effects from ketone bodies through reduced consumption of oxygen, likely extends to nondiabetic patients (80).

SGLT2i elevates hematocrit through several pathways. First, because of an increased delivery of glucose to the transporters downstream in the medullary segments, a compensatory upregulation of SGLT1 likely occurs, resulting in increased oxygen demand, and thus a risk of hypoxia in this area (35). It should be noted that that the sodium reabsorption independent of glucose by transporters in the inner stripe of the outer medullar may contribute to outer medullary hypoxia. SGLT2i may induce erythropoietin due to increased hypoxia at the corticomedullary junction, related to the translocation of tubular transport from cortical segments to medullary thick ascending limbs (101). Studies showed that this mechanism may stimulate erythropoietin (EPO) producing fibroblasts (25, 77). Animal studies support the described mechanisms for example determination of the intrarenal distribution of tissue oxygenation following SGLTi with the use of oxygen microelectrodes (102). Gullaksen et al. used Blood Oxygenation Level Dependent Magnetic Resonance Imaging (BOLD-MRI) for calculating an apparent relaxation rate in patients with diabetes mellitus type 2. Administration of empagliflocin for 32 weeks changed cortical oxygenation from 23.6 Hz (95%CI, 23.1-24.1) to 23.3 Hz (965% CI, 2.5-24.0; p=0.231) (103). Administration of empagliflozin for 32 weeks reduced medullary oxygenation from 24.5 Hz (95%CI, 23.9-24.9) to 25.4 Hz (95%CI, 24.7-26.2; p= 0.003; where higher apparent relaxation rate corresponds to a lower oxygenation) (103). They indicated that apparent relaxation rate is not a direct measure of oxygenation and is dependent on deoxyhemoglobin concentrations (103). Furthermore, they observed that compared to baseline values the estimated marginal means of both hematocrit and plasma erythropoietin increased after administration of empagliflozin. They concluded that on the contrary to their initial hypothesis empagliflozin reduced medullary kidney oxygenation and hypothesized that the hypoxia generated by empagliflozin stimulates erythropoietin synthesis which may mediate kidney protection (103). The complex association linking the impact of SGLT2i to proteinuria at the glomerular and tubular level, to renal oxygenation, and on the progression to chronic kidney disease has recently been reviewed in-depth by Heyman et al. (104). Increased transglomerular hydraulic pressure induces hyperfiltration and increases the albumin leak across the filtration barrier (104). The reduction of transglomerular hydraulic pressure and hyperfiltration by Angiotensin Receptor Blockers or SGLT2i may attenuate or prevent albuminuria for the long-term (104).

Second, SGLT2 inhibition likely reduces the effective circulating volume. Heerspink et al. showed that administration of a SGLT2i increased hematocrit, hemoglobin, and transiently elevated reticulocyte count and erythropoietin concentrations (25). They suggest that both volume constriction and increased red blood cell mass may contribute to that effect.

Increased ketogenesis as well as elevated hematocrit may improve renal tissue oxygenation, by compensating imbalances between oxygen consumption and oxygen delivery (76). Because tubular sodium reabsorption largely contributes to energy utilization and thus oxygen consumption, reduced proximal tubule transporter activity and thus workload may be important (43). Laursen et al. were able to demonstrate that a single high dose of the SGLT2i dapagliflozin improved renal cortical oxygenation within six hours in patients with type 1 DM and albuminuria. They did not observe changes in renal blood flow or blood oxygen saturation. Therefore, they suggested that the improvement was due to a reduction in tubular workload (26). Liu et al. showed that treatment with the SGLT2i ertugliflozin was associated with sustained lowering of kidney injury molecule 1, a biomarker specific to proximal tubules in patients with type 2 DM (27). This biomarker is sensitive and specific to kidney injury, with increased secretion from tubular cells to the urine under hypoxic conditions, and it correlates well with the onset and progression of CKD (27, 105).

### 3.4 Antiinflammatory effects of SGLT2i

Fibrosis is likely the results of dysfunctional autophagia in combination with inflammation, and it is characterized by fewer number of functional nephrons. Autophagia is typically suppressed in states of nutrient overabundance (44). The beneficial effect of SGLT2i on fibrosis may be secondary to oxidative and organellar stress (99). In diabetic patients, autophagic proteins are decreased, and the levels correlate with the stage of CKD (106, 107). Autophagia is important for the clearance of damaged proteins and organelles, and thus the prevention of inflammation (106). Inflammation has been proposed as being a prominent feature of CKD (45, 108, 109). Treatment with SGLT2 inhibitors may contribute to increased autophagia, decreased inflammation and thereby prevent fibrosis through their actions regarding ketone bodies. Ketone bodies upregulates transcription factors of the starvation like stage which likely promote autophagia (46). Because ketone bodies are not fully utilized, they may also work as inhibitors of histone deacetylases and inflammasomes (47). The improved tissue oxygenation may also contribute to reduced inflammation and fibrosis, which may be enhanced by hypoxia inducible transcription factors (38). Bessho reported that the SGLT2i luseoglifozin inhibited hypoxia-induced hypoxia inducible factor-1α protein expression in human renal proximal tubular epithelial cells (110). Dekkers et al. showed a reduction in urinary markers of inflammation, including kidney injury molecule, in diabetic patients upon SGLT2 inhibition (28). The reduction correlated positively with the reduction in albuminuria and eGFR (28). Due to several beneficial effects of SGLT2i this therapy has been introduced together with other agents to maximally slow CKD progression.

### 3.5 Regulation of the transporter

Most studies in humans reported an increased expression of SGLT2 in diabetic patients compared to healthy controls. Wang et al. found an increased expression of SGLT2 mRNA and protein in biopsies from patients with type 2 DM and CKD compared to healthy controls (29). Rahmoune et al. also found increased expression of SGLT2 mRNA and protein in proximal tubular cells from urine samples from patients with type 2 DM compared to healthy controls (30). In contrast, Solini et al. observed reduced expression of SGLT2 mRNA and protein in tissue from nephrectomies obtained from patients with type 2 DM and renal carcinoma (31). These conflicting results may be due to methodological differences including the type of tissue obtained, methods of measurement or sample bias. It could also be a consequence of differences regarding the diseases. Furthermore, the CKD stage may also be a crucial parameter since expression of mRNA from tubular cells have been reported to correlate with GFR (32).

Regarding the expression of SGLT2 in nondiabetic patients, results have been conflicting. On one hand, Raisekeran et al. reported decreased expression of SGLT2 mRNA in biopsies from patients with obesity related FSGS compared to control kidney donors (21). This reduction may reflect proximal tubule cell injury and the absence of stimulatory hyperglycemic milieu. On the other hand, Sridhar et al. detected increased expression of SGLT2 mRNA in biopsies from control kidney donors and patients with nondiabetic nephropathy involving different subtypes of GN, compared to patients with diabetic nephropathy (32). They observed no differences across GN subtypes. Renal biopsies are infrequent in diabetic patients with CKD and reserved for advanced proteinuria or severe insufficiency, thus decreased SGLT2 mRNA could reflect the more advanced stages of CKD in these patients.

Until recently, RAAS blockade using ACEi and ARB have been the cornerstones for the treatment of diabetic and nondiabetic patients with CKD. Several trials have demonstrated the efficiency of these treatments compared to placebo. It should be noted that the event rates were much higher in these trials, which yielded a number needed to treat ranging from 4 to 23 (111–115). In most trials investigating SGLT2i, the patients received SGLT2i on top of RAAS blocking agents. In the placebo-controlled trials using SGLT2i, the number needed to treat ranged from 9 to 93 (5, 7, 9, 116–118). Thus, comparing the different trial designs may indicate that blocking both, RAAS and SGLT2, may be necessary to prevent progression of kidney disease (**Table 5**). Different mechanisms observed in patients with diabetes mellitus type 1, diabetes mellitus type 2, and nondiabetic chronic kidney disease are summarized in **Table 6**.

**Table 5:** Effectiveness of treatment with SGLT2 inhibitors compared to placebo as observed in major clinical outcome studies in diabetic and nondiabetic patients.

Author and title	Publication year Study design Population Follow-up period	Event description	Treatment groups: events and total subjects	Event rate Numbers needed to treat (NNT)
***Heerspink et al.:*** **Dapagliflozin in Patients with Chronic Kidney Disease** (5)	2020 RCT Diabetic and nondiabetic nephropathy 2.4 years	Composite endpoint	Dapagliflozin: 197 events out of 2152 subjects Placebo: 312 events out of 2152 subjects	Dapagliflozin: 0.092 Placebo: 0.145 NNT= 19
***Wiviott et al.:*** **Dapagliflozin and Cardiovascular Outcomes in Type 2 Diabetes** (7)	2019 RCT Diabetic nephropathy 4.2 years	Composite endpoint	Dapagliflozin: 127 events out of 8582 subjects Placebo: 238 events out of 8578 subjects	Dapagliflozin: 0.015 Placebo: 0.028 NNT= 77
***Perkovic et al.:*** **Canagliflozin and Renal Outcomes in Type 2 Diabetes and Nephropathy** (9)	2019 RCT Diabetic nephropathy 2.6 years	Doubling of serum creatinine	Canagliflozin: 118 events out of 2202 subjects Placebo: 188 events out of 2199 subjects	Canagliflozin: 0.054 Placebo: 0.085 NNT= 31
***Lewis et al.:*** **The effect of angiotensin-converting-enzyme inhibition on diabetic nephropathy** (110)	1993 RCT Diabetic nephropathy 3 years	Doubling of serum creatinine	Captopril: 25 events out of 207 subjects Placebo: 43 events out of 202 subjects	Captopril: 0.121 Placebo: 0.213 NNT= 11
***Maschio et al.:*** **Effect of the angiotensin-converting-enzyme inhibitor benazepril on the progression of chronic renal insufficiency** (111)	1996 RCT Nondiabetic nephropathy 3 years	Doubling of serum creatinine and dialysis	Benzepril: 31 events out of 300 subjects Placebo: 57 events out of 283 subjects	Benzepril: 0.103 Placebo: 0.201 NNT= 10
***GISEN group:*** **Randomised placebo-controlled trial of effect of ramipril on decline in glomerular filtration rate and risk of terminal renal failure in proteinuric, non-diabetic nephropathy** (112)	1997 RCT Nondiabetic nephropathy 3.3 years	Doubling of serum creatinine	Ramipril: 18 events out of 78 subjects Placebo: 40 events out of 88 subjects	Ramipril: 0.231 Placebo: 0.455 NNT= 4
***Brenner et al.:*** **Effects of losartan on renal and cardiovascular outcomes in patients with type 2 diabetes and nephropathy** (113)	2001 RCT Diabetic nephropathy 3.4 years	Doubling of serum creatinine	Losartan: 162 events out of 751 subjects Placebo: 198 events out of 762 subjects	Losartan: 0.216 Placebo: 0.260 NNT= 23
***Lewis et al.:*** **Renoprotective effect of the angiotensin-receptor antagonist irbesartan in patients with nephropathy due to type 2 diabetes** (114)	2001 RCT Diabetic nephropathy 2.6 years	Doubling of serum creatinine	Irbesartan: 98 events out of 579 subjects Placebo: 135 events out of 569 subjects	Irbesartan: 0.169 Placebo: 0.237 NNT= 15
***Hou et al.:*** **Efficacy and safety of benazepril for advanced chronic renal insufficiency** (115)	2006 RCT Nondiabetic nephropathy 3.4 years	Doubling of serum creatinine, end stage renal disease, death	Benzepril: 44 events out of 108 subjects Placebo: 65 events out of 107 subjects	Benzepril: 0.407 Placebo: 0.607 NNT= 5
***Wanner et al.:*** **Empagliflozin and Progression of Kidney Disease in Type 2 Diabetes** (116)	2016 RCT Diabetic nephropathy 3.2 years	Doubling of serum creatinine	Empagliflozin: 70 events out of 4645 subjects Placebo: 60 events out of 2323 subjects	Empagliflozin: 0.015 Placebo: 0.026 NNT= 93
***Wheeler et al.:*** **A pre-specified analysis of the DAPA-CKD trial demonstrates the effects of dapagliflozin on major adverse kidney events in patients with IgA nephropathy** (117)	2021 RCT IgA nephropathy 2.4 years	Composite endpoint	Dapagliflozin: 6 events out of 137 subjects Placebo: 20 events out of 133 subjects	Dapagliflozin: 0.044 Placebo: 0.150 NNT= 9
***Wheeler et al.:*** **Safety and efficacy of dapagliflozin in patients with focal segmental glomerulosclerosis: A prespecified analysis of the DAPA-CKD trial** (118)	2021 RCT FSGS 2.4 years	Composite endpoint	Dapagliflozin: 4 events out of 45 subjects Placebo: 7 events out of 59 subjects	Dapagliflozin: 0.089 Placebo: 0.119 NNT= 34

For comparison, the effectiveness of treatment with ACEi and ARB are also shown.

**Table 6:** Different mechanisms observed in patients with diabetic mellitus type 1, diabetes mellitus type 2, and nondiabetic chronic kidney disease.

Reference	Proposed different mechanisms
***Liu et al.:*** **Cardiorenal protection with SGLT2 inhibitors in patients with diabetes mellitus: from biomarkers to clinical outcomes in heart failure and diabetic kidney disease** (36)	Afferent vasoconstriction and efferent vasodilatation. Inflammation markers in patients with type 2 DM treated with SGLT2 inhibitors.
**Yoshimoto et al.: Effects of sodium-glucose cotransporter 2 inhibitors on urinary excretion of intact and total angiotensinogen in patients with type 2 diabetes** (45)	Angiotensin converting enzyme 2 causing stimulation of the vasodilating and anti-inflammatory alternative pathways, i.e., different balance between angiotensin I (promoting vasoconstriction) and angiotensin 1-7 (promoting vasodilatation)
***Hou et al.:*** **Molecular Mechanisms of SGLT2 Inhibitor on Cardiorenal Protection** (59)	Arteriole constriction by adenosine and efferent arteriole dilatation by prostaglandins
***Schnell et al.:*** **Comparison of mechanisms and transferability of outcomes of SGLT2 inhibition between type 1 and type 2 diabetes** (62)	Angiotensin converting enzyme 2 causing stimulation of the vasodilating and anti-inflammatory alternative pathways.
***Oguz et al.:*** **Inhibition of sodium glucose cotransporter 2 to slow the progression of chronic kidney disease** (82)	Restoration of tubuloglomerular feedback including ketogenesis, renal tissue oxygenation, inflammation, fibrosis

## 4 Discussion

This narrative review is written in accordance to the principles stated by Green et al. (119). This review highlights the beneficial effects of sodium glucose cotransporter type 2 inhibitors in patines with kidney diseases. The effects can be attributed to systemic, hemodynamic, and metabolic effects. SGLT2i show beneficial effects on blood pressure and restoration of tubuloglomerular feedback.

## 5 Conclusions

Nephroprotection offered by SGLT2i can be attributed to systemic, hemodynamic, and metabolic mechanisms, with restoration of tubuloglomerular feedback likely being most important. Diabetic and nondiabetic patients with CKD share common features which are targeted by SGLT2i. These include similar steps in the pathogenesis, namely glomerular hyperfiltration, as well as final common pathways involving imbalances in tissue oxygenation, inflammation, and fibrosis. The main mechanisms underlying the nephroprotective effects in diabetic patients seem transferable to nondiabetic patients. According to current literature, the underlying mediators may be different. The underlying disease may affect the cellular expression of SGLT2 and may therefore determine the benefit from the SGLT2i treatment.

## Author contributions

ES: Methodology, Validation, Writing – original draft, Writing – review & editing, Data curation, Formal analysis, Investigation. MT: Methodology, Validation, Writing – original draft, Writing – review & editing, Conceptualization, Supervision, Visualization.
